# A secondary analysis to determine variations of dental arch measurements with age and gender among Ugandans

**DOI:** 10.1186/s13104-015-1411-6

**Published:** 2015-09-10

**Authors:** Hilda Okori, Pricilla S. Apolot, Erisa Mwaka, Gerald Tumusiime, William Buwembo, Ian G. Munabi

**Affiliations:** Department of Human Anatomy, School of Biomedical Sciences, Makerere University College of Health Sciences, New Mulago Hospital Complex, P.O.Box 7072, Kampala, Uganda, East Africa

**Keywords:** Dental arch, Dimensions, Age, Anatomy, Forensic odontology, Gender

## Abstract

**Background:**

Dental arch dimensions are useful in dental practice and in forensic odontology. Local data is essential because ethnic differences exist in dental arch dimensions. In the Ugandan population no studies had been done on dental arch dimensions. The objective of the current study was to determine the variations in dental arch dimensions with age and gender in a sample of dental casts from the Ugandan population.

**Method:**

This was a secondary analysis of dental casts previously prepared using mandibular and maxillary arch impressions of 220 children (85 boys and 135 girls) aged 12–17 years recruited from schools in Kampala, Uganda. Dental arch dimensions for the maxilla and mandibular casts were taken using a digital vernier calliper. The data was analysed using the means based independent samples *t* test to obtain the descriptive statistics with regression analysis being used to obtain the regression coefficients and constants using STATA 12.

**Results:**

The overall maxillary dimensions were significantly smaller in females than males by 1.50 mm (95 % CI −2.91 to −0.09, P = 0.04), controlling for age group. The overall dimensions of the mandible were also smaller in younger participants, though this was not statistically significant.

**Conclusion:**

From this study we observed significant differences in arch dimensions between males and females that are of forensic value for this population. There is need for more study of the differences in arch dimensions with age using a larger and more age diverse study population.

## Background

Dental arch dimensions are used in dentistry to guide the provision of accurate orthodontic diagnosis, and in forensic medicine to aid the identification of human remains [1, 2]. Observations from literature show that different human ethnic groups display unique dental arch characteristics and measurements [3]. As an example, comparison between Caucasian and Japanese mandibular arch forms revealed Caucasians to have smaller inter-canine width and inter-molar width in all three Angles mal-occlusion classes [4]. Meaning that different ethnic groups have different arch dimensions [5]. In many developing countries there is an increase in number of patients with dental problems that require orthodontic interventions [6–8]. These orthodontic interventions (diagnosis, treatment plan and prognosis) are all based on knowledge of population estimates of these dental arch dimensions [9]. A clinician would therefore need a representative set of population derived dental arch dimensions to provide accurate orthodontic treatment to their clients or to identify a client in the case of forensic studies.

Dental arch dimensions have been observed to vary with both age and gender. With respect to age, the dental arch dimensions change a lot during the periods of intense growth as is seen in childhood and the teenage years only to lessen during adulthood [9–12]. Carter et al., found a decrease with age in the upper and lower inter-canine distance [9]. The observations in these periods of mixed dentition are the result of tooth movement and growth of the supporting bone [13]. The growth and development of dental arch dimensions also differ with respect to gender. Bishara found that above age 25 years, upper and lower inter-canine distance increases in females and only lower inter canine distance increase in males [10]. The differences in dental arch dimensions are influenced by different factors, the most important of which is the genetic component [10].

The Ugandan population is made up of a large diversity of ethnic groupings exposed to various challenges, like the high prevalence of malnutrition; as such one would expect a set of unique dental arch dimension measurements [14]. The uniqueness of these measurements when compared with those from other populations could be attributed to differences in environment, nutrition, systemic health and individual variations [2]. To our knowledge, there has been no study to determine the dental arch dimensions of the Ugandan population. The aim of the current study therefore was to determine the variations in dental arch dimensions with age and gender in a sample of dental casts from the Ugandan population.

## Methods

This was a secondary analysis of mandibular and maxillary dental casts from a sample of Ugandans obtained as part of a previous study [15], whose aim was to predict the width of un-erupted incisors, canines and premolars. These casts were obtained from dental impressions made in five secondary schools within a radius of 6 km from Makerere University College of Health Sciences as previously described [15]. The schools were from peri-urban Kampala the Capital city of Uganda and most of the parents of the participants were middle income earners, hence the children were from an urban setting. In summary, five study schools were randomly selected from a list of 35 schools using computer generated numbers. The study sample comprised of school children (n = 220) aged 12–17 years who had all their permanent teeth erupted with the exception of the third molar, and were all of Angles class I occlusion. Many [16–18] previous authors show differences in arch dimensions though some [19] do not agree and indicate that: [no statistically significant differences in arch width are found between the different classes analyzed (there are only slight differences between classes), except in the case of mandibular inter-canine width, which is smaller in Class I than in Class II division 1, and maxillary inter-premolar width, which is smaller in Class II division 1 than in Class I]. So in the current study we took the position of not pooling the different classes together.

All the participating children were Ugandans of African descent with every fifth child being systematically randomly selected to participate in the study from a line of single gender children. A total of 232 children were selected and examined to determine their dentition status and those with crowded, spaced, malformed or missing teeth (except third molar), inter-proximal caries, inter-proximal restorations or history of orthodontic treatment were excluded. Twelve (12) children were excluded because they did not have normal contact points on proximal tooth surfaces, leaving a sample of 220 children (85 boys and 135 girls). Nine (2:7, M:F) of the excluded children were of age group (12–14), while 3 (1:2, M:F) were of age group (15–17). Dental casts were made using dental stone (Gypsano LLC, Fujariah, United Arab Emirates) from each child’s dental impression as described before [15]. A summary of the participant population is provided in Table 1.

**Table 1:** Descriptive statistics of the study population

Variable	Gender		
	Female	Male	
Age group 1 (12–14 years)	53 (67.9 %)	25 (32.1 %)	78 (100 %)
Age group 2 (15–17 years)	81 (56.6 %)	62 (43.4 %)	143 (100 %)
Total	134 (60.6 %)	87 (39.4 %)	221 (100 %)

Dental arch dimensions of width and length were taken by one examiner using a digital vernier calliper (Mitutoyo, Southampton, UK). The following measurements were made: total length of dental arch, length of the anterior segment of the arch, length of the posterior segment of the arch, inter-canine width, inter-first premolar width, inter-first molar width, for both the maxilla and mandibular casts [1]. Double data entry by two researchers followed by validation was done using EPIDATA 3.1 software. The data was eventually exported to STATA 12 (STATA Corp LP, TX, USA) for analysis using the means based independent samples t test to obtain the descriptive statistics. Ordinary regression analysis was used to obtain the univariable regression coefficients and constants. Multilevel regression analysis using the gllamm function in STATA 12, was used to obtain the regression coefficients for the overall combined repeated mandibular and maxillary measurements. During the analysis, information from the measurements was tagged by a unique study identifier number for each cast and linked to the raw data from the previous study to obtain socio demographic characteristics of each participant. All the measurements in the tables were reported in millimetres in the format (y = (g) x + constant), where (g) is the regression coefficient, to support prediction. The level of significance was set at 0.05. Permission to use the archived dental casts was obtained from the Department of Anatomy, School of Biomedical Sciences, Makerere University College of Health Sciences, where they were previously kept.

## Results

Table 2, provides a summary of the differences in the means of the study measurements with respect to the age group and gender of the individual participants. With respect to age, the maxillary: inter-canine distance (P = 0.03), second inter-premolar distance (P = 0.02), first molar distance (P = 0.01) and mandibular total arch length (P = 0.03) were the only measurements with significant differences in the means of the respective study age groups. For gender, significant differences were observed in the following maxillary measurements: inter-canine distance (P < 0.01), second inter-premolar distance (P < 0.01), first molar distance (P < 0.01) and total arch length (P < 0.01) were larger in male participants. Equally significant larger measurements were observed in each of the above mandibular participants’ measurements and the anterior segment length (P < 0.01), for male participants. All the other measurement had no significant difference in their respective means across the two study groups.

**Table 2:** Comparison of different dental arch measurements by age and gender

Measurement	Overall mean (SD)	Age group 1 mean (SD)	Age group 2 mean (SD)	T test (P value)	Female mean (SD)	Male mean (SD)	T test (P value)
Maxillary							
Inter-canine distance	35.93 (2.86)	35.36 (3.10)	36.24 (2.67)	−2.19 (0.03)	35.30 (2.86)	36.90 (2.60)	4.19 (<0.01)
Second inter-premolar distance	42.64 (3.12)	41.98 (2.98)	43.00 (3.14)	−2.33 (0.02)	41.84 (2.98)	43.86 (2.94)	4.95 (<0.01)
First molar distance	48.30 (3.46)	47.52 (3.27)	48.73 (3.50)	−2.5 (0.01)	47.32 (3.36)	49.82 (3.07)	5.55 (<0.01)
Total arch length	32.68 (3.01)	32.32 (2.82)	32.89 (3.10)	−1.34 (0.18)	32.24 (3.13)	33.39 (2.67)	2.8 (0.01)
Anterior segment length	13.36 (2.07)	13.16 (2.20)	13.50 (1.99)	−1.16 (0.25)	13.21 (2.11)	13.64 (2.00)	1.51 (0.13)
Posterior segment length	20.00 (6.55)	19.16 (1.56)	20.46 (8.07)	−1.41 (0.16)	19.70 (6.34)	20.47 (6.89)	0.85 (0.40)
Mandibular							
Inter-canine distance	27.06 (2.71)	26.72 (3.01)	27.24 (2.53)	−1.35 (0.18)	13.20 (2.11)	27.86 (2.67)	3.58 (<0.01)
Second inter-premolar distance	36.55 (3.28)	36.04 (3.51)	36.84 (3.13)	−1.73 (0.09)	35.60 (2.94)	38.04 (3.25)	5.76 (<0.01)
First molar distance	41.72 (4.14)	41.14 (3.11)	42.05 (4.62)	−1.53 (0.13)	40.72 (4.47)	43.19 (3.09)	4.35 (<0.01)
Total arch length	29.35 (3.09)	28.73 (2.91)	27.72 (3.15)	−2.23 (0.03)	29.88 (3.22)	29.00 (2.95)	2.01 (0.05)
Anterior segment length	9.26 (2.43)	9.08 (1.93)	9.36 (2.67)	−0.82 (0.42)	10.04 (2.97)	8.76 (1.86)	3.97 (<0.01)
Posterior segment length	25.57 (18.91)	22.37 (13.67)	27.31 (21.06)	−1.86 (0.06)	26.62 (20.28)	23.95 (16.55)	−1.02 (0.31)

The significant differences observed above are further emphasised in the regression analysis for age (Table 3) and gender (Table 4). In Table 3, only the regression coefficients for inter-canine distance (0.88, 95 % CI 0.09–1.67, P = 0.03), second inter-premolar distance (1.02, 95 % CI 0.16–1.88, P = 0.02) and first molar distance (1.21, 95 % CI 0.26–2.16, P = 0.01) for the maxillary measurements with only the mandibular measurement: for total arch length (0.99, 95 % CI 0.12–1.86, P = 0.03), significantly increased in moving from age group 1 to age group 2.

**Table 3:** Regression analysis showing variation in dental arch dimensions with Age

Measurement (mm)	Regression coefficient (95 % CI)	P value	Constant (95 % CI)
Maxillary (overall)	0.96 (−0.47 to 2.40)	0.19	31.56 (30.41 to 32.72)
Inter-canine distance	0.88 (0.09 to 1.67)	0.03	35.36 (34.73 to 36.00)
Second inter-premolar distance	1.02 (0.16 to 1.88)	0.02	41.98 (41.30 to 42.68)
First molar distance	1.21 (0.26 to 2.16)	0.01	47.52 (46.76 to 48.29)
Total arch length	0.57 (−0.26 to 1.40)	0.18	32.32 (31.65 to 32.99)
Anterior segment length	0.34 (−0.24 to 0.92)	0.25	13.16 (12.70 to 13.62)
Posterior segment length	1.30 (−0.52 to 3.12)	0.16	19.16 (17.70 to 20.62)
Mandibular (overall)	1.20 (−0.29 to 2.70)	0.12	27.30 (26.10 to 28.50)
Inter-canine distance	0.52 (−0.24 to 1.27)	0.18	26.72 (26.12 to 27.33)
Second inter-premolar distance	0.80 (−0.11 to 1.71)	0.09	36.04 (35.31 to 36.77)
First molar distance	0.92 (−0.26 to 2.10)	0.13	41.14 (40.20 to 42.08)
Total arch length	0.99 (0.12 to 1.86)	0.03	28.73 (28.03 to 29.42)
Anterior segment length	0.28 (−0.4 to 0.95)	0.42	9.08 (8.54 to 9.63)
Posterior segment length	4.94 (−0.31 to 10.18)	0.07	22.37 (18.15 to 26.59)

**Table 4:** Regression analysis showing variation in dental arch dimensions with Gender

Measurement	Regression coefficient (95 % CI)	P value	Constant (95 % CI)
Maxillary (overall)	−1.50 (−2.91 to −0.09)	0.04	33.10 (32.00 to 34.19)
Inter-canine distance	−1.60 (−2.35 to −0.85)	<0.01	36.90 (36.31 to 37.48)
Second inter-premolar distance	−2.02 (−2.83 to −1.22)	<0.01	43.86 (43.23 to 44.49)
First molar distance	−2.50 (−3.38 to −1.61)	<0.01	49.82 (49.12 to 50.51)
Total arch length	−1.15 (−1.96 to −0.34)	0.01	33.39 (32.75 to 34.02)
Anterior segment length	−0.44 (−1.00 to 0.13)	0.13	13.64 (13.20 to 14.08)
Posterior segment length	−0.78 (−2.57 to 1.02)	0.40	20.47 (19.07 to 21.87)
Mandibular (overall)	−1.05 (−2.52 to 0.42)	0.16	28.71 (27.56 to 29.85)
Inter-canine distance	−1.31 (−2.04 to −0.59)	<0.01	27.86 (27.29 to 28.42)
Second inter-premolar distance	−2.44 (−3.28 to −1.61)	<0.01	38.04 (37.38 to 38.68)
First molar distance	−2.47 (−3.59 to −1.35)	<0.01	43.19 (42.32 to 44.05)
Total arch length	−0.88 (−1.74 to −0.02)	0.05	29.88 (29.21 to 30.55)
Anterior segment length	−1.29 (−1.93 to −0.65)	<0.01	10.04 (9.55 to 10.54)
Posterior segment length	2.68 (−2.48 to 7.83)	0.31	23.94 (19.92 to 27.97)

Table 4 shows that the overall maxillary dimensions were significantly smaller in females than males by 1.50 mm (95 % CI −2.91 to −0.09, P = 0.04), controlling for age group. The overall dimensions of the mandible were also smaller in females, though this was not significant. Significant variables were: maxillary inter canine distance, total arch length, posterior segment length and anterior segment length. Mandibular variables are all significant except the total arch length.

## Discussion

We set out to determine the variations in dental arch dimensions with age and gender in a sample of dental casts from the Ugandan population. We found that overall the dental arch measurements were significantly smaller in females compared to male participants. Also there were significant increases in some of the measurements with increasing age.

The observed significant differences in the dental arch measurements with respect to gender as observed in Table 4 are useful in dental practice and more importantly applicable to forensic odontology [2]. It is important to note that these measurements are influenced by individual genetics leading to observed racial differences [5]. To our knowledge no such studies had been done on the Ugandan population, this is the first study providing this local data. In this study we found significant differences in the maxillary measurements: inter-canine distance (P < 0.01), second inter-premolar distance (P < 0.01), first molar distance (P < 0.01) and total arch length (P < 0.01) which were larger in male participants. This observation confirms earlier reports [2, 20] showing that male participants had larger dimensions, while on the other hand contradicts observations made by other investigators who report for example that both genders had nearly similar inter-canine distance [21]. One explanation for this observation may be that the population studied are of different age ranges which would affect the dental arch dimensions. It is also possible that participants of different classifications of mal-occlusion could have been pooled together in the different studies. In this study the analysis for the general dimensions of the maxilla and the mandible were done controlling for the age of the participant. This makes the observations in Table 4 especially useful for forensic deontology in these settings.

With respect to age only the regression coefficients for: inter-canine distance (P = 0.03), second inter-premolar distance (P = 0.02) and first molar distance (P = 0.01) for the maxillary measurements with only the mandibular measurement: for total arch length (P = 0.03), significantly increased with age. It is possible that the majority of the age related changes in dental arch dimensions may have been missed due to the grouping of the participant’s ages and the actual age range of the study population. From literature, it has been observed that significant changes in dental arch dimensions are associated with development of the permanent dentition from the age of 6 years (first molar) to 12 years (second molar) and finally 18 years (third molar) [9–12]. It is also important to note that the dental arch dimensions will also tend to differ between different participants of Angles classification [22, 23], this is an additional source of variation that must be taken care of in such a study. So in this study, while we only included participants of Angles class I, it is possible that we may have missed the majority of changes in dental arch dimensions given our older study population, older than the mixed dentition. The measurements in Table 3; still have relevance in forensic odontology and orthodontics. In orthodontics they can be used for prediction and making appropriate adjustments.

The limitations of this study were that the primary data collected had the age of the participants clustered in groups at the time of measurement. Despite this short coming the information generated by the study is important to the practice of forensic medicine and orthodontics.

## Conclusions

From this study the differences in arch dimensions between males and females are of forensic value for this population. There is need for more study of the differences in arch dimensions with age using a larger and more age diverse study population.
